# The Impact of Psychological Distress on Cervical Cancer

**DOI:** 10.3390/cancers15041100

**Published:** 2023-02-09

**Authors:** Chen-Ta Wu, Lu-Ting Chiu

**Affiliations:** 1 Department of Radiation Oncology, Hualien Tzu Chi Hospital, Buddhist Tzu Chi Medical Foundation, Hualien 970473, Taiwan; 2 Management Office for Health Data, China Medical University Hospital, Taichung 404327, Taiwan; 3 College of Medicine, China Medical University, Taichung 406040, Taiwan

**Keywords:** mood disorders, gynecologic malignancy, depression, bipolar, cervix

## Abstract

**Simple Summary:**

Mood disorders including depression and bipolar were associated with an increased hazard ratio of cervical cancer. The cumulative incidence of cervical cancer was significantly higher in participants with mood disorders than in the non-mood-disorder cohort. Thus, mood disorders could potentially increase the risk of developing subsequent cervical cancer.

**Abstract:**

Objective: Psychological distress is considered a factor for cancer development. However, the impact of mood disorders (depression and bipolar) on the development of cervical cancer remains uncertain. We conducted a nationwide population-based retrospective cohort study to investigate the association between mood disorders and the subsequent risk of developing cervical cancer. Methods: A total of 138,130 participants’ profiles between 2000 and 2012 were extracted from the National Health Insurance Research Database and subdivided into a mood-disorder cohort (27,626 participants) and a non-mood-disorder cohort (110,504 participants). Cohorts were propensity-matched for a 1:4 ratio according to age and index year. The Cox proportional hazards regression model was utilized for assessing cervical cancer risk between cohorts. Results: Kaplan–Meier analysis revealed that the mood-disorder cohort had a higher cumulative incidence of cervical cancer. The mood-disorder cohort was also associated with an increased risk of cervical cancer after adjustments for potential confounders. Subgroup analysis revealed a negative impact of mood disorders on cervical cancer, especially in the 30–50 years and white-collar groups. Conclusions: Our findings demonstrated that mood disorders were associated with an increased risk of cervical cancer development, which provide helpful information for clinical strategies to reduce the incidence of cervical cancer in this vulnerable population.

## 1. Introduction

It is estimated that around half a million women are affected by cervical cancer annually worldwide, and the majority of cases are from developing countries. According to the World Health Organization, cervical cancer ranks as the fourth-most-common malignancy in women. In Taiwan, cervical cancer was ranked eighth place in 2013–2016 with an incidence rate of 8.72 per 100,000 individuals, accounting for 3.12% of all incident cases [1]. Although a slowly decreasing trend in cervical cancer was reported worldwide from 1990 to 2019 [2], cervical cancer remains a substantial health problem for women globally. As such, much interest has been paid to investigate the risk factors associated with the development of cervical cancer [3,4,5]. These risk factors can be categorized as human papillomavirus (HPV)-related and non-HPV-related factors; the latter includes socioeconomic status, cigarette smoking, alcohol, and genetics [3,4,5]. 

Mental diseases have been recognized as a contributing risk factor for cancer development [6,7,8]. The possible mechanisms underlying the impact of mental diseases on cancer development may rest on various physiological, psychological, and genetic grounds [8,9,10]. For example, mental diseases may dysregulate immune function, which contributes to cancer development [11]. Females with mental diseases have an under-screening for cervical cancer, leading to an increased risk of this type of cancer [12]. Moreover, mental diseases and cancer may share a common genetic contribution [13]. To this end, although mood disorders are among the most common mental diseases [14,15,16], their negative impact on the development of cancer is still controversial. Several meta-analyses demonstrated that mental diseases were significantly associated with increased risks of overall cancer and certain specific types of cancer [6,7], while others showed patients with mental diseases had no increased risk [17] or even a reduced risk [18] of developing cancer. Similarly, some individual cohort studies reported that the overall incidence of cancer was increased in the mental-disease group compared to the comparison group [19,20,21], whereas others reported null effect of mental diseases on cancer incidence [22].

It is well known that women have a higher lifetime prevalence of mood disorders than men do [23]. Some cohort studies showed an increased risk of cervical cancer among female patients with schizophrenia [19] or bipolar [20], while others demonstrated the opposite or null result in female patients with depression [20] or anxiety [22]. Accordingly, the impact of mood disorders on the subsequent development of cervical cancer remains uncertain. While the impact of mental diseases on the development of cancer has been well described in the Western literature, it has been shown that Asian women are more likely to be diagnosed with cervical cancer than white women [24]. Furthermore, there are racial and ethnic differences in lifetime risk of mental disorders among Asians and people of other races/ethnicities in the USA [25]. As such, this topic warrants investigation using data from Taiwan because the prevalence of mood disorder among Taiwanese women has increased by approximately 20% during a 10-year period [26] and cervical cancer is a substantial health problem for Taiwanese women [1].

The objective of this investigation was to evaluate the impact of mood disorders including depression and bipolar on the subsequent development of cervical cancer in Taiwan. We hypothesized that patients with mood disorders have an increased risk of the development of cervical cancer. To test this hypothesis, we conducted a retrospective cohort study with a follow-up period of around 7 years in the mood-disorder group and the non-mood-disorder group. The approach of using a retrospective design allowed us to have a large sample size for the propensity-matched analysis, which minimized confounding and other sources of bias arising from the use of observational data. Since age, comorbidity, urbanization level, and employment category (white collar, blue collar, or other) are influencing factors for the development of cervical cancer in patients with mental diseases or in the general population [17,18,19,20,21,22,27,28], we further performed subgroup analyses according to these demographic and socioeconomic factors.

## 2. Methods

### 2.1. Data Source

The Taiwanese National Health Insurance (NHI) program, which was initiated in March 1995, covers 99% of the 23.7 million of residents of Taiwan. The National Health Research Institutes (NHIR) receives insurance claims data from the National Health Insurance Administration (NHIA) and compiles them into the National Health Insurance Research Database (NHIRD). The NHIRD has detailed medical records for each patient, including basic demographic variables of sex and age and disease diagnosis codes from both outpatient and inpatient records [29]. The NHIRD encrypts patients’ personal information including personal identification numbers, birth date, and names to protect privacy [29,30]. In the present study, we used the Longitudinal Health Insurance Database 2000 (LHID2000), a subset of the NHIRD specifically constructed for research purposes, containing 1,000,000 beneficiaries (approximately 5% of Taiwan’s population) randomly sampled from the NHIRD [29]. The data sampling from the LHID2000 dataset is representative of the whole population during this time frame. The reason for using the LHID2000 is because Taiwan’s National Health Research Institutes has restricted the amount of data requested by researchers to ≤10% of Taiwan’s population [29]. This restriction is one of the protective strategies for data confidentiality. Diagnostic codes were based on the International Classification of Diseases, 9th Revision, Clinical Modification (ICD-9-CM). The validity and accuracy of ICD-9-CM codes have been demonstrated in previous studies [29]. This study was approved by the Research Ethics Committee of China Medical University and Hospital in Taiwan (CMUH-104-REC2-115-CR-4). For this retrospective study, informed consent was waived according to the institutional guidelines.

### 2.2. Study Population

**Figure 1:**
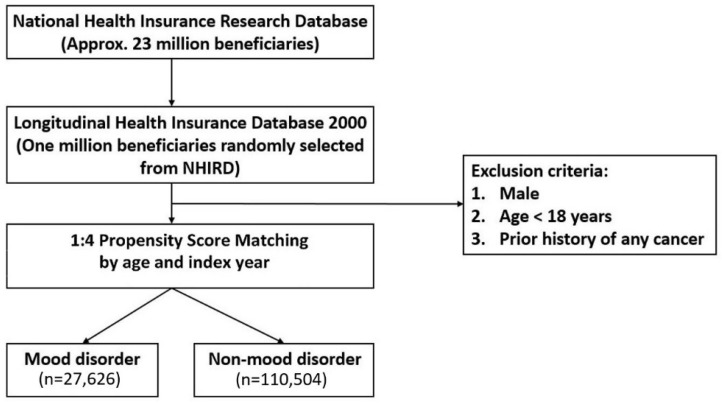
Study flowchart. The flowchart shows the subject selection in this study.

We constructed a population-based retrospective cohort study to determine whether patients with mood disorders are more susceptible to developing cervical cancer. The mood-disorder cohort included women aged above 18 years who received a diagnosis of mood disorders (depression and bipolar) with at least two outpatient visits or at least one hospitalization record from 2000 to 2012 (Figure 1). Participants in the non-mood-disorder cohort were defined as participants without mood disorders. The diagnostic codes for mood disorders used to identify patients in this study are listed below. For depressive disorder, we used ICD-9-CM code 296.2 (major depressive disorder single episode), code 296.3 (major depressive disorder recurrent episode), code 300.4 (dysthymic disorder), and code 311 (depressive disorder, not elsewhere classified). For bipolar disorder, we used code 296.0 (manic disorder, single manic episode), code 296.1 (manic disorder, recurrent episode), code 296.4 (bipolar I disorder, most recent episode (or current) manic), code 296.5 (bipolar I disorder, most recent episode (or current) depressed), code 296.6 (bipolar I disorder, most recent episode (or current) mixed), code 296.7 (bipolar I disorder, most recent episode (or current) unspecified), code 296.8 (other affective psychoses), code 296.80 (bipolar disorder, unspecified), and code 296.89 (bipolar II disorder). The date of the first diagnosis of mood disorders was defined as the index date. Women aged above 18 years who had never received a diagnosis of mood disorders during the study period were randomly selected as the comparison cohort and propensity-matched with the mood-disorder cohort at a 4:1 ratio according to age and index year. We excluded participants with a history of any cancer (ICD-9-CM 140-208) before the index date. The main outcome was a new diagnosis of cervical cancer (ICD-9-CM 180.9 and 233.1) during the follow-up period. All participants in this study were followed from the index date until being diagnosed with cervical cancer and censored because of the loss to follow-up, withdrawal from insurance, or at the end of 2013. Baseline comorbidities before the index date including diabetes mellitus (ICD-9-CM 250), hypertension (ICD-9-CM 401-40 and A26), hyperlipidemia (ICD-9-CM 272), cardiovascular disease (ICD-9-CM 402, 410-414, 420-429, 430-438), chronic kidney disease (ICD-9-CM 580-589, A350), dementia (ICD-9-CM 290 and 294), and sexually transmitted diseases (ICD-9-CM 042, 054.1, 078.11, 091-098, 099.5 and 131) were investigated in this study. We also considered utilization of Pap smears in this study, and Pap smear density was calculated as the number of Pap smears per year during the follow-up period. The participants’ demographic characteristics were collected from both cohorts including age, urbanization level, employment category, and the follow-up duration.

### 2.3. Statistical Analysis

The chi-square test and the independent *t*-test were used to test the difference between the two cohorts for categorical variables and continuous variables, respectively. The Cox proportional hazards regression model was performed to calculate the hazard ratio (HR) with 95% confidence intervals (CI) for cervical cancer risk between the mood-disorder and non-mood-disorder cohorts. The adjusted HR was obtained after adjustment for potential confounders. We estimated the cumulative risk of cervical cancer in the mood-disorder and non-mood-disorder cohorts with the Kaplan–Meier method, and the differences were examined using a log-rank test. The risks of cervical cancer in the mood-disorder and non-mood-disorder cohorts were calculated with stratification by age and comorbidities. We managed and analyzed the data with SAS 9.4 software (SAS Institute, Cary, NC, USA) and drew the cumulative incidence curve with R software (R Foundation for Statistical computing, Vienna, Austria). Two-tailed *p* values of <0.05 were considered to be statistically significant.

## 3. Results

Baseline participant characteristics between the mood-disorder and non-mood-disorder cohorts are shown in Table 1. A total of 27,626 participants with mood disorders and 110,504 participants without mood disorders were included in this study cohort. Both cohorts have a similar age distribution. For the mood-disorder cohort, the mean age was 44.34 ± 16.21 years while the mean age was 44.38 ± 16.16 years in the non-mood-disorder cohort. The mean follow-up periods were 7.11 ± 3.71 years in the mood-disorder cohort and 7.08 ± 3.73 years in the non-mood-disorder cohort. Compared to the non-mood-disorder cohort, the prevalence of all comorbidities and Pap smears were significantly higher in the mood-disorder cohort (*p* < 0.0001). There were significant differences in urbanization level and employment category between the two cohorts (*p* < 0.0001). After propensity matching with age and index year, the mood-disorder and non-mood-disorder cohorts appeared to be well balanced, except that two comorbidities and Pap smear density had between-group differences (Table 1).

Table 2 shows the cervical cancer incidence rates for the mood-disorder and non-mood-disorder cohorts were 5.98 and 3.49 per 10,000 person-years, respectively. After adjustment for all the covariates, the risk of cervical cancer was 1.76-fold higher in the mood-disorder cohort than in the non-mood-disorder cohort (95% CI = 1.40–2.19). Compared with participants aged <30 years, the risk of cervical cancer was 1.60-fold higher in those aged 30 to 50 years (95% CI = 1.21–2.12) and 1.80 fold higher in those aged above 50 years (95% CI = 1.30–2.50). The risk of cervical cancer was 1.36-fold higher in blue-collar workers (95% CI = 1.06–1.69) than others. As to comorbidities, in the multi-variable adjusted model, the risk of cervical cancer among participants with and without comorbidities was non-significant. Thus, the Cox regression analysis revealed that the mood-disorder cohort had a significantly higher HR for developing cervical cancer compared to the non-mood-disorder group (Table 2). In addition, except mood disorders as a risk factor, analyses of other covariates demonstrated that participants aged >30 years, living in low levels of urbanization, and with a blue-collar occupation had a significantly higher HR for developing cervical cancer compared to participants aged <30 years, living in highest level of urbanization, and with a white-collar occupation, respectively (Table 2).

Further analyses of the cervical cancer risk stratified according to age and comorbidities in the mood-disorder and non-mood-disorder cohorts are shown in Table 3. In participants aged 30 to 50 years, the mood-disorder cohort had a 2.27-fold increased risk of cervical cancer compared to the non-mood-disorder cohort. The mood-disorder cohort had a significantly increased risk of cervical cancer than the non-mood-disorder cohort among patients without comorbidities. Thus, these subgroup analyses indicated that the mood-disorder-related increased risk of developing cervical cancer was apparent in participants with age 30–50 years, without comorbidities analyzed in this study, living in the highest level of urbanization, and with white-collar occupations (Table 3).

**Figure 2:**
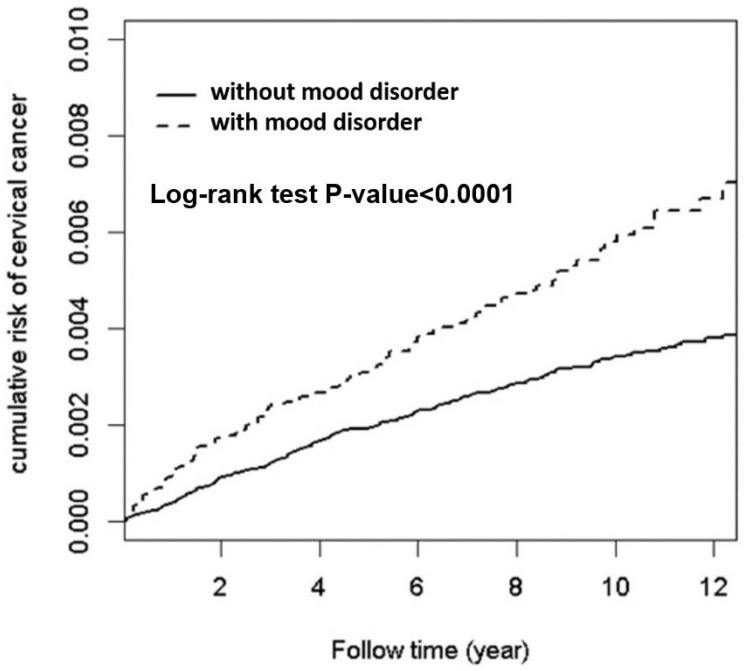
Kaplan–Meier survival curves compared the cumulative risk of cervical cancer between cohorts with and without mood disorders. The KM survival curves showed a higher cumulative risk of cervical cancer in the cohort with mood disorders compared to the cohort without mood disorders.

We also observed that a significant relationship was only exhibited among the highest urbanization level and white-collar groups. The Kaplan–Meier method was applied for cervical cancer patient survival curve analysis between the mood-disorder cohort and the non-mood-disorder cohort (Figure 2). The cumulative risk of cervical cancer was significantly higher in participants with a mood disorder than in the non-mood-disorder cohort (log-rank test *p* < 0.0001).

## 4. Discussion

In this study, we conducted a large-scale nationwide population-based retrospective cohort investigation to evaluate the association between mood disorders and the subsequent risk of developing cervical cancer. Our results suggest that mood disorders are a negative prognostic factor for the development of cervical cancer in Taiwan. Except the present study, only one investigation [20] showed that bipolar, but not depression, was associated with the development of cervical cancer. The authors used a psychiatric inpatient medical claims database and reported that the estimated number of cervical cancer cases in their study patients with bipolar was higher than the national incidence rates reported on the website. Our study used a different approach to explore this topic and showed that the HR of developing cervical cancer was increased in the mood-disorder cohort compared to the non-mood-disorder cohort. Apart from depression and bipolar, one cohort study showed an increased risk of cervical cancer among patients with schizophrenia [19], while the other demonstrated a reduced risk in patients with anxiety [22]. Our results give new evidence to support the notion that mental diseases are contributing risk factors for the subsequent development of cervical cancer.

The exact link between mood disorders and development of cervical cancer remains unclear. Several possible physiological, psychological, and genetic mechanisms [8,9,10] may be involved. With regard to physiological mechanisms, mental diseases may dysregulate several physiological functions leading to dysfunction of immune function, which in turn contributes to cancer development [10,11,31]. For example, it has been suggested that stress and depression can foster tumor progression by inhibition of the expression of class-I and class-II major histocompatibility complex molecules and by reducing NK activity [11]. Indeed, mental diseases are associated with decreased cytotoxic T cell and natural killer cell activities that are critical for the immune surveillance of tumors [11]. A previous study [32] reported that stressful negative life events were associated with a lower T cytotoxic/suppressor cell percentage and natural killer cell cytotoxicity in Black women and suggested that these events may lead to immune decrements, poor control over HPV infection, and increased vulnerability of developing cervical cancer. With regard to psychological mechanisms, several psychological factors have been found to contribute to a low Pap screening participation in women with mental diseases [33]. In fact, it has been reported that women with mental diseases are more than five times less likely to receive adequate Pap screening compared with the general population and this under-screening may predispose them to an increased risk of cervical cancer [12]. The innate psychological reasons for a link between mental diseases and cervical cancer may be due to negative emotional states, poor ego defense mechanisms, coping skills, a sad disposition following loss, and locus of control [8,34]. With regard to genetic mechanisms, mental diseases and cancer may share a common genetic contribution and patients with mental diseases may have a genetic predisposition to cancer development [13]. Two recent studies [35,36] have identified several shared loci associated with risks of mental diseases and cancer. Collectively, these multiple mechanisms may contribute to the negative impact of mood disorders on the development of cervical cancer we observed in this study.

Age is a major influencing factor for the development of cervical cancer. Recent global statistical analysis showed that the incidence of cervical cancer starts rising after the age of 25 years, reaching a maximum incidence around the age of 40 years in the highest-resource countries, whereas rates continued to rise markedly up to ages 55–69 years in lower-resource countries [37]. In this study, the mood-disorder and non-mood-disorder cohorts had similar frequencies in various age groups because this covariate was propensity-matched. We found that the age groups of 30–50 years and of 50 years or more had a higher risk of developing cervical cancer compared to the age group of <30 years (Table 2), a finding that is consistent with previous observations [37]. Interestingly, our subgroup analyses indicated that the negative impact of mood disorders on the development of cervical cancer was observed only in the age group of 30–50 years (Table 3). Lin et al. [19] reported that female schizophrenia patients aged 20–60 years had a higher overall incidence rate of cancer compared to the same age group in the general population. Hung et al. [20] reported that patients with bipolar aged <80 years and patients with depression aged 20–70 years had a higher overall incidence rate of cancer than the national cancer incidence rate. It appears that the vulnerability of patients with mental diseases to cancer is age-dependent. 

Socioeconomic status is another major influencing factor for the development of cervical cancer [38,39]. Low-income, less-educated, working-class populations and populations with a low level of urbanization have been found less likely to receive Pap smear screening than their counterparts [39,40,41]. Several socioeconomic factors have been found to contribute to the low Pap screening participation in women with mental diseases [33]. In this study, we found that the level of urbanization had null effect on the risk of cervical cancer. However, female blue-collar workers had an increased risk of cervical cancer compared to female white-collar workers (Table 2). Of note, our subgroup analyses indicated that the negative impact of mood disorders on the development of cervical cancer was observed only in participants living in the highest level of urbanization and with a white-collar occupation (Table 3). These findings seem to be contradictory to the concept regarding the negative impacts of low income, less education, and low level of urbanization on Pap screening participation. However, participants with mood disorders who have white-collar occupations living in the highest level of urbanization may have high stress and a busy/unstable life style, both of which may potentiate the negative impact of mood disorders on the development of cervical cancer. 

It is known that the presence of concurrent non-psychiatric diseases in an individual diagnosed with a mood disorder is associated with a more complex disease presentation and management [16]. We found that participants with comorbidities did not have an increased risk of developing cervical cancer compared to participants without comorbidities (Table 2). However, our subgroup analyses indicated that the negative impact of mood disorders on the development of cervical cancer was observed in participants without comorbidities (Table 3). This may be due to the small sample size of participants with comorbidities leading to low statistical power.

The strength of this study is that our nationwide population-based model offers large numbers of subjects over significant periods of time, which can be very informative. However, several limitations must be considered in this study. One major limitation is the lack of complete information regarding the lifestyle or behavioral factors of patients in the NHIRD database. Therefore, our study was unable to fully adjust for factors such as smoking habit, marital status, educational level, income status, and obstetric history, which are unavailable from the NHI dataset. Furthermore, family history is an important covariate that should be considered. Family relationships can be identified in the original NHIRD given that only spouses and blood relatives could be qualified dependents of the insured individuals covered by the Taiwan NHI [42]. However, the identification of family lineages was incomplete in this study because we used the LHID2000. Second, the retrospective nature our study is subject to several biases, including data collection from the database and the inherent differences between patients in the mood-disorder and non-mood-disorder cohorts. We believe that these biases were minimized by the study design of the propensity-matched analyses. 

However, it is still possible that there were unmeasured differences between these two cohorts that may account for our observed results, for example, an individual may have developed other psychiatric disorders first, and then was diagnosed with mood disorders. Third, utilizing only ICD-9-CM Diagnosis Codes to identify patients with mood disorders may not represent the real situation due to cultural differences, where Asian patients tend to be more reluctant to seek professional psychiatric assistance. The actual number of psychiatric cases could be underestimated. As a result, patients with depression and bipolar were pooled into a single cohort in this study due to their relatively limited sample size. Fourth, the findings from our subgroup analyses should be interpreted with caution as stratification results in limited statistical power. For the same reason, we did not study the impact of mood disorders in subgroups of depressive disorder and bipolar disorder.

## 5. Conclusions

In conclusion, our results demonstrated that mood disorders including depression and bipolar were associated with an increased risk of cervical cancer, particularly in the age group of 30–50 years and in participants with white-collar occupations. Our findings provide the observation that mood disorders could potentially increase the risk of developing subsequent cervical cancer. Further prospective research is warranted to validate the findings from this study.

## Figures and Tables

**Table 1:** Baseline characteristics of participants.

Characteristics	Mood Disorders				*p*-Value	Standardized Mean Difference
	No		Yes			
	(*n* = 110,504)		(*n* = 27,626)			
	*n*	%	*n*	%		
**Age**					1.00	0.000
<30	24,728	22.38	6182	22.38		
30–50	47,336	42.84	11,834	42.84		
>50	38,440	34.79	9610	34.79		
Mean ± SD	44.34 ± 16.21		44.38 ± 16.16		0.82	0.002
**Comorbidity**						
Cardiovascular disease	17,731	16.05	9691	35.08	<0.0001	0.191
Chronic kidney disease	4946	4.48	2302	8.33	<0.0001	0.069
Dementia	669	0.61	717	2.60	<0.0001	0.080
Diabetes mellitus	9668	8.75	3566	12.91	<0.0001	0.057
Hypertension	20,133	18.22	7387	26.74	<0.0001	0.085
Hyperlipidemia	13,438	12.16	5810	21.03	<0.0001	0.103
Sexually transmitted disease	1377	1.25	731	2.65	<0.0001	0.046
**^a^ Pap smear**	8298	7.51	3395	12.29	<0.0001	0.069
**^b^ Pap smear density**	0.02 ± 0.08		0.03 ± 0.11		<0.0001	0.115
**Urbanization**					<0.0001	0.023
1 (high)	36,020	32.62	9312	33.71		
2	32,957	29.84	8560	31.00		
3	18,864	17.08	4152	15.04		
4 (low)	22,593	20.46	5591	20.25		
**Employment category**					<0.0001	0.035
White collar	61,279	55.45	14,423	52.21		
Blue collar	40,730	36.86	10,497	38.00		
Others	8495	7.69	2706	9.80		
**Follow-up time, years**	7.11 ± 3.71		7.08 ± 3.73		0.28	0.008

Data shown as *n* (%) or mean ± SD; ^a^ Pap smear: patients who had a Pap smear during follow-up period; ^b^ Pap smear density: number of Pap smears during follow-up period/follow-up year; standardized mean difference > 0.1 was considered as imbalance in the two groups.

**Table 2:** Cox proportional hazard regression model measured hazard ratios and 95% CI of cervical cancer diagnosis associated with mood disorders and other covariates.

Variables	Cervical Cancer (*n* = 391)			Crude HR (95% CI)	Adjusted HR (95% CI)
	Event	PY	IR		
**Mood disorder**					
No	274	785,733	3.49	1 (reference)	1 (reference)
Yes	117	195,682	5.98	1.71 (1.38–2.13) ***	1.76 (1.40–2.19) ***
**Age**					
<30	67	236,420	2.83	1 (reference)	1 (reference)
30–50	197	439,509	4.48	1.58 (1.19–2.08) **	1.60 (1.21–2.12) **
>50	127	305,485	4.16	1.42 (1.06–1.92) *	1.80(1.30–2.50) ***
**Comorbidity**					
Cardiovascular disease					
No	305	812,268	3.75	1 (reference)	1 (reference)
Yes	86	169,146	5.08	1.32 (0.96–1.67)	0.97 (0.71–1.34)
Chronic kidney disease					
No	373	940,197	3.97	1 (reference)	1 (reference)
Yes	18	41,217	4.37	1.06 (0.66–1.70)	1.09 (0.66–1.80)
Dementia					
No	389	975,300	3.99	1 (reference)	1 (reference)
Yes	2	6114	3.27	0.76 (0.19–3.06)	0.70 (0.17–2.86)
Diabetes mellitus					
No	360	904,163	3.98	1 (reference)	1 (reference)
Yes	31	77,252	4.01	0.97 (0.68–1.41)	1.03 (0.67–1.57)
Hypertension					
No	316	808,702	3.91	1 (reference)	1 (reference)
Yes	75	172,713	4.34	1.09 (0.84–1.40)	0.87 (0.62–1.02)
Hyperlipidemia					
No	348	869,127	4	1 (reference)	1 (reference)
Yes	43	112,288	3.83	0.96 (0.67–1.26)	0.86 (0.59–1.25)
Sexually transmitted disease					
No	383	969,414	3.95	1 (reference)	1 (reference)
Yes	8	12,001	6.67	1.61 (0.80–3.25)	1.08 (0.53–2.20)
**Urbanization**					
1 (high)	107	324,278	3.3	1 (reference)	1 (reference)
2	122	295,210	4.13	1.25 (0.96–1.62)	1.21 (0.93–1.57)
3	66	162,496	4.06	1.23 (0.90–1.67)	1.31 (0.96–1.78)
4 (low)	95	198,842	4.78	1.44 (1.09–1.90) **	1.13 (0.84–1.51)
**Employment category**					
White collar	193	540,999	3.57	1 (reference)	1 (reference)
Blue collar	166	362,011	4.59	1.28 (1.04–1.58) *	1.36 (1.06–1.69) *
Others	32	78,404	4.08	1.14 (0.78–1.66)	1.21 (0.83–1.76)

* *p* < 0.05, ** *p* < 0.01, *** *p* < 0.001; Abbreviations: PY, person-years; IR, incidence rate, per 10,000 person-years; HR, hazard ratio; CI, confidence interval; HR adjusted for patient age, cardiovascular disease, chronic kidney disease, dementia, diabetes mellitus, hypertension, hyperlipidemia, sexually transmitted disease, urbanization, and employment category. Models adjusted for Pap smear density listed in Table 1.

**Table 3:** Incidence and hazard ratio of cervical cancer stratified by age and other covariates between patients with and without mood disorder.

Variables	Mood Disorder						Compared to without Mood Disorders	
	No			Yes			Crude HR	Adjusted HR
	Event	PY	IR	Event	PY	IR	(95% CI)	(95% CI)
**Cervical cancer**	274	785,733	3.49	117	195,682	5.98	1.71 (1.38–2.13) ***	1.76 (1.40–2.19) ***
**Age**								
<30	46	188,954	2.43	21	47,465	4.42	1.81	1.36
							(1.08–3.04) *	(0.79–2.37)
30–50	128	352,008	3.64	69	87,500	7.89	2.17	2.27
							(1.62–2.90) ***	(1.67–3.08) ***
>50	100	244,769	4.09	27	60,715	4.45	1.08	0.96
							(0.71–1.66)	(0.68–1.46)
**Comorbidity**								
Cardiovascular disease								
No	233	679,815	3.43	82	132,452	6.19	1.89	1.68
							(1.47–2.44) ***	(1.29–2.18) ***
Yes	51	105,916	4.82	35	63,229	5.54	1.17	1.26
							(0.76–1.80)	(0.80–1.99)
Chronic kidney disease								
No	266	758,455	3.51	107	181,742	5.89	1.68	1.73
							(1.34–2.10) ***	(1.37–2.18) ***
Yes	8	27,277	2.93	10	13,939	7.17	2.54	2.43
							(0.99–6.44)	(0.90–6.55)
Dementia								
No	274	783,190	3.49	115	192,109	5.99	1.71	1.75
							(1.38–2.13) ***	(1.40–2.20) ***
Yes	0	2541	-	2	3572	5.59		
Diabetes mellitus								
No	257	730,114	3.52	103	174,048	5.92	1.68	1.71
							(1.34–2.11) ***	(1.35–2.17) ***
Yes	17	55,618	3.06	14	21,633	6.47	2.13	1.74
							(0.95–4.33)	(0.80–3.76)
Hypertension								
No	224	660,599	3.39	92	148,101	6.21	1.83	1.63
							(1.43–2.33) ***	(1.26–2.10) ***
Yes	50	125,133	3.99	25	47,580	5.25	1.32	1.46
							(0.82–2.14)	(0.88–2.42)
Hyperlipidemia								
No	247	708,286	3.48	101	160,840	6.28	1.80	1.84
							(1.43–2.27) ***	(1.45–2.34) ***
Yes	27	77,446	3.49	16	34,841	4.69	1.31	1.10
							(0.71–2.44)	(0.57–2.13)
Sexually transmitted disease								
No	1270	777,953	16.32	113	191,459	5.91	1.70	1.75
							(1.36–2.12) ***	(1.39–2.20) ***
Yes	4	7779	5.14	4	4221	9.48	1.86	2.14
							(0.47–7.45)	(0.47–9.59)
**Urbanization**								
1 (high)	72	258,020	2.79	35	66,257	5.28	1.89	1.71
							(1.26–2.83) **	(1.12–2.61) *
2	85	234,058	3.63	37	61,151	6.05	1.67	1.34
							(1.13–2.45) **	(0.89–2.04)
3	48	133,542	3.59	18	28,953	6.22	1.73	1.62
							(1.00–2.96) *	(0.92–2.83)
4 (low)	69	159,576	4.32	26	39,265	6.62	1.53	1.84
							(0.98–2.41)	(1.15–2.94)
**Employment category**								
White collar	134	437,725	3.06	59	103,273	5.71	1.87	1.98
							(1.37–2.53) ***	(1.44–2.72) ***
Blue collar	119	288,451	4.13	47	73,559	6.39	1.54	1.32
							(1.10–2.17) *	(0.92–1.89)
Others	21	59,555	3.53	11	18,848	5.84	1.65	1.26
							(0.79–3.42)	(0.56–2.80)

* *p* < 0.05, ** *p* < 0.01, *** *p* < 0.001; Abbreviations: PY, person-years; IR, incidence rate, per 10,000 person-years; HR, hazard ratio; CI, confidence interval; HR adjusted for patient age, cardiovascular disease, chronic kidney disease, dementia, diabetes mellitus, hypertension, hyperlipidemia, sexually transmitted disease, urbanization, and employment category. Models adjusted for Pap smear density listed in Table 1.

## Data Availability

Data are available from the National Health Insurance Research Database (NHIRD) published by the Taiwan National Health Insurance (NHI) Bureau. Due to legal restrictions imposed by the government of Taiwan in relation to the “Personal Information Protection Act”, data cannot be made publicly available. Requests for data can be sent as a formal proposal to the NHIRD (http://nhird.nhri.org.tw (accessed on 25 January 2023)).
